# Combinatorial pathway enzyme engineering and host engineering overcomes pyruvate overflow and enhances overproduction of *N*-acetylglucosamine in *Bacillus subtilis*

**DOI:** 10.1186/s12934-018-1049-x

**Published:** 2019-01-04

**Authors:** Wenlong Ma, Yanfeng Liu, Xueqin Lv, Jianghua Li, Guocheng Du, Long Liu

**Affiliations:** 1 Key Laboratory of Carbohydrate Chemistry and Biotechnology, Ministry of Education, Jiangnan University, Wuxi, 214122 China; 2 Key Laboratory of Industrial Biotechnology, Ministry of Education, Jiangnan University, Wuxi, 214122 China

**Keywords:** *Bacillus subtilis*, *N*-Acetylglucosamine, Glucosamine-6-phosphate *N*-acetyltransferase, Pyruvate, Overflow, Urease

## Abstract

**Background:**

Glucosamine-6-phosphate *N*-acetyltransferase (GNA1) is the key enzyme that causes overproduction of *N*-acetylglucosamine in *Bacillus subtilis*. Previously, we increased GlcNAc production by promoting the expression of GNA1 from *Caenorhabditis elegans* (*Ce*GNA1) in an engineered *B. subtilis* strain BSGN12. In this strain overflow metabolism to by-products acetoin and acetate had been blocked by mutations, however pyruvate accumulated as an overflow metabolite. Although overexpression of *Ce*GNA1 drove carbon flux from pyruvate to the GlcNAc synthesis pathway and decreased pyruvate accumulation, the residual pyruvate reduced the intracellular pH, resulting in inhibited *Ce*GNA1 activity and limited GlcNAc production.

**Results:**

In this study, we attempted to further overcome pyruvate overflow by enzyme engineering and host engineering for enhanced GlcNAc production. To this end, the key enzyme *Ce*GNA1 was evolved through error-prone PCR under pyruvate stress to enhance its catalytic activity. Then, the urease from *Bacillus paralicheniformis* was expressed intracellularly to neutralize the intracellular pH, making it more robust in growth and more efficient in GlcNAc production. It was found that the activity of mutant *Ce*GNA1 increased by 11.5% at pH 6.5–7.5, with the catalytic efficiency increasing by 27.5% to 1.25 s^−1^ µM^−1^. Modulated expression of urease increased the intracellular pH from 6.0 to 6.8. The final engineered strain BSGN13 overcame pyruvate overflow, produced 25.6 g/L GlcNAc with a yield of 0.43 g GlcNAc/g glucose in a shake flask fermentation and produced 82.5 g/L GlcNAc with a yield of 0.39 g GlcNAc/g glucose by fed-batch fermentation, which was 1.7- and 1.2-times, respectively, of the yield achieved previously.

**Conclusions:**

This study highlights a strategy that combines pathway enzyme engineering and host engineering to resolve overflow metabolism in *B. subtilis* for the overproduction of GlcNAc. By means of modulated expression of urease reduced pyruvate burden, conferred bacterial survival fitness, and enhanced GlcNAc production, all of which improved our understanding of co-regulation of cell growth and metabolism to construct more efficient *B. subtilis* cell factories.

**Electronic supplementary material:**

The online version of this article (10.1186/s12934-018-1049-x) contains supplementary material, which is available to authorized users.

## Background

**Fig. 1:**
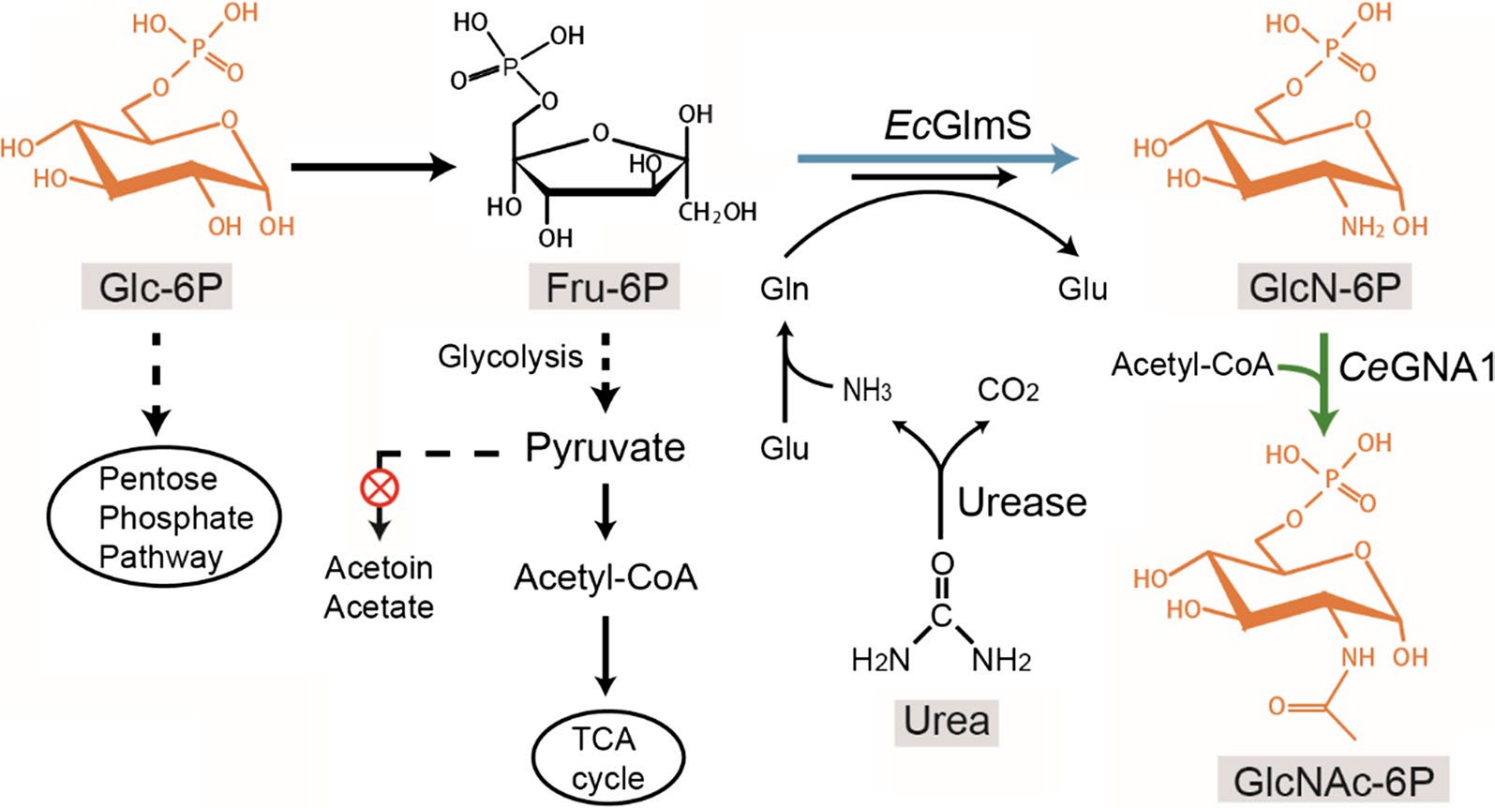
Schematic overview of engineering *Bacillus subtilis* for GlcNAc production. *Ec*GlmS: glucosamine-6-phosphate synthase from *Escherichia coli*; *Ce*GNA1: glucosamine-6-phosphate *N*-acetyltransferase from *Caenorhabditis elegans*; Glc-6P: glucose-6-phosphate; Fru-6P: fructose-6-phosphate; GlcN-6P: glucosamine-6-phosphate; GlcNAc-6P: *N*-acetylglucosamine-6-phosphate; Glu: glutamate; Gln: glutamine

*N*-Acetylglucosamine (GlcNAc), a functional monosaccharide with many specific bioactivities, has received considerable attention for its commercial applications in the biomedical, food, and chemical industries [1, 2]. In the pathway toward biosynthesis of GlcNAc, glucosamine-6-phosphate *N*-acetyltransferase from *Caenorhabditis elegans* (*Ce*GNA1 [EC 2.3.1.4]), catalyzing the formation of GlcNAc-6-phosphate (GlcNAc-6P) by the acetylation of GlcN-6-phosphate (GlcN-6P) using the cofactor Ac-CoA, holds a key position (Fig. 1) [3, 4].

In previous studies, using 5′-terminus fusion engineering, we promoted heterogenous expression of *Ce*GNA1 and glutamine-fructose-6-phosphate aminotransferase from *Escherichia coli* (*Ec*GlmS) in the recombinant *B. subtilis* strain BSGN12. Specifically, the expression level of *Ce*GNA1 was enhanced via fusing epitope tag cMyc to the 5′-terminus of *Ce*GNA1 gene and ribosome binding site (RBS) sequence engineering. Next, the expression level of *Ec*GlmS was enhanced by fusing mRNA stabilizer Δ*ermC*+14/7 downstream of the strong P_*veg*_ promoter and inserting it in the *Bacillus subtilis* chromosome at the *als* locus. The enhanced expression of *Ce*GNA1 consumed AcCoA for GlcNAc-6-phosphate synthesis, which further facilitated pyruvate conversion to AcCoA and decreased pyruvate accumulation. The enhanced expression of *Ec*GlmS consumed fructose-6-phosphate for GlcNAc-6-phosphate synthesis, competed with glycolysis and decreased pyruvate formation. Also, the enhanced expression of *Ce*GNA1 and *Ec*GlmS decreased pyruvate accumulation and promoted GlcNAc production in the recombinant *B. subtilis* strain BSGN12. However, the fact that overflow of metabolic by-products acetoin and acetate had been blocked by mutations in *alsRSD* and *ackA*, meant that pyruvate accumulated as an overflow metabolite in this strain (Fig. 1) [5, 6].

Though overexpression of *Ce*GNA1 and *Ec*GlmS decreased pyruvate accumulation for GlcNAc-6P synthesis, there was still a small amount of pyruvate (~ 3.5 g/L) that accumulated in the broth during fermentation. Herein, we found that the residual pyruvate acidified the extracellular pH (pH_ex_) and intracellular pH (pH_in_), with the lowest pH_ex_ and pH_in_ being 5.7 and 6.0 for BSGN12 during fermentation. The pH_in_ critically affects bacterial cell physiology, such as protein synthesis and enzyme activity [7–10]. The activity of *Ce*GNA1 is pH-dependent with an optimum pH of 8.2, which is similar to other GNA1 homologues that generally function in alkaline conditions (pH 7.4–9.7), thus it is crucial to maintain intracellular pH homeostasis for the enhanced activity of *Ce*GNA1 and improved production of GlcNAc [4].

In this study, for increasing GlcNAc production, error-prone PCR (Ep-PCR) based directed evolution of *Ce*GNA1 was conducted during pyruvate stress to enhance its catalytic activity, and one mutant *Ce*GNA1-Q155V/C158G, whose activity in the pH 6.5–7.5 increased by 11.5% with the catalytic efficiency increasing by 27.5% to 1.25 s^−1^ μM^−1^, was obtained. Shake flask fermentation showed that the evolved *Ce*GNA1-Q155V/C158G increased the GlcNAc titer by 11.3% to 20.6 g/L. Next, the urease from *Bacillus paralicheniformis* was expressed under the control of exponential phase-dependent promoter (P_*hag*_) to neutralize the pH_in_. Expression of urease enhanced urea utilization and increased the pH_in_ from 6.0 to 6.8, making it more robust in growth and more efficient in GlcNAc production, with the GlcNAc titer and yield reaching 25.6 g/L and 0.43 g GlcNAc/g glucose, respectively. In a 3-L fermenter, the final strain overcame pyruvate overflow and produced 82.5 g/L GlcNAc with a yield of 0.39 g GlcNAc/g glucose, which was 1.7- and 1.2-times that of the control. The data highlight the importance of pathway enzyme engineering and host engineering in regulating activities of key enzyme *Ce*GNA1 to overcome pyruvate overflow and efficiently produce GlcNAc in engineered *B. subtili*s factories.

## Materials and methods

### Strains, plasmids, and culture conditions

The bacterial strains and plasmids used in this study are listed in Table 1. The primers are listed in the Additional file 1: Table S1. BSGN12 (Δ*nagP*Δ*gamP*Δ*gamA*Δ*nagA*Δ*nagB*Δ*ldh*ΔptaΔ*ackA::lox72*, Δ*alsRSD::*Pveg-Δ*ermC*+14/7A-*ecglm*), which secreted pyruvate into the medium during fermentation, was used as the host strain [6]. During the construction of the strains and plasmids, all strains were grown at 37 °C in standard Luria–Bertani broth (LB) (10 g/L tryptone, 5 g/L yeast extract, 10 g/L NaCl) or LB agar plates, with an appropriate concentration of antibiotics used for selection (100 μg/mL ampicillin, 25 μg/mL kanamycin, or 30 μg/mL zeocin).

**Table 1:** Strains and plasmids used in this study

	Characteristics	References
*Strains*		
BSGN5	*B. subtilis* 168 derivate, Δ*nagP*Δ*gamP*Δ*gamA*Δ*nagA*Δ*nagB*Δ*ldh::lox72*	[30]
BSGN10	*B. subtilis* 168 derivate, Δ*nagP*Δ*gamP*Δ*gamA*Δ*nagA*Δ*nagB*Δ*ldh*Δ*alsRSD::lox72*	[5]
BSGN12	*B. subtilis* 168 derivate, Δ*nagP*Δ*gamP*Δ*gamA*Δ*nagA*Δ*nagB*Δ*ldh*ΔptaΔ*ackA::lox72*, Δ*alsRSD::*Pveg-Δ*ermC*+14/7A-*ecglmS*	[6]
BSGN12-P_*veg*_-urease	BSGN12 derivate, expression of urease from *Bacillus paralicheniformis* under the control of promoter P_*veg*_	This study
BSGN12-P_*xylA*_-urease	BSGN12 derivate, expression of urease from *Bacillus paralicheniformis* under the control of promoter P_*xylA*_	This study
BSGN12-P_*abrB*_-urease	BSGN12 derivate, expression of urease from *Bacillus paralicheniformis* under the control of promoter P_*abrB*_	This study
BSGN13	BSGN12 derivate, expression of urease from *Bacillus paralicheniformis* under the control of promoter P_*hag*_	This study
BSGN12-P_*ffh*_-urease	BSGN12 derivate, expression of urease from *Bacillus paralicheniformis* under the control of promoter P_*ffh*_	This study
BSGN12-P_*licH*_-urease	BSGN12 derivate, expression of urease from *Bacillus paralicheniformis* under the control of promoter P_*licH*_	This study
*Plasmids*		This study
p7Z6	pMD18-T containing *lox71*-*zeo*-*lox66* cassette	[19]
pTSC	Em^r^Amp^r^; temperature sensitive in *B. subtilis*	[19]
pP_*43*_-cMyc (M-Rm)-*CeGNA1*	key enzyme *Ce*GNA1 expressing vector	[6]
pP_*43*_-cMyc (M-Rm)-*CeGNA1*-Q155V/C158G	pP_*43*_-cMyc (M-Rm)-*CeGNA1* derivate, with 155Gln and 158Cys of *CeGNA1* mutated to 155Val and 158Gly, respectively	This study
pP_*43*_-6His-*Ce*GNA1	pP_*43*_NMK derivate with *CeGNA1* cloned	[6]
pCold-*CeGNA1*	pCold III derivate, containing wild type *Ce*GNA1, with His tag fused to the N terminal	This study
pCold-*CeGNA1*-Q155V/C158G	pCold III derivate, containing mutant *Ce*GNA1-Q155V/C158G, with His tag fused to the N terminal	This study
pP_*veg*_*EcGlmS*-1	pUC57-Amp derivate, containing the synthesized expression cassette, terminator I-P_*veg*_-TSS-RBS_0_-terminator II	[6]
pStop1622	Amp^r^, Tet^r^, *E. coli*-*B. megaterium* shuttle vector	[31]

During shake flask and fed-batch fermentations, the following fermentation medium was used: urea, 5 g/L; (NH_4_)_2_SO_4_ 6 g/L; yeast extract, 12 g/L; tryptone, 6 g/L; K_2_HPO_4_·3H_2_O, 18.75 g/L; MgSO_4_, 3 g/L; FeSO_4_·7H_2_O, 0.06 g/L; CaCl_2_, 0.06 g/L; and NiCl_2_·6H_2_O, 0.12 g/L. Glucose was sterilized separately, and added to the shake flask to a final concentration of 60 g/L. Xylose (final concentration, 10 g/L) was added to the fermentation medium when the optical density at 600 nm (OD_600_) reached 0.6 to induce the expression of urease controlled by P_*xylA*_ promoter.

### Detection of pH_in_

The pH_in_ of cells were assayed using a pH-sensitive fluorescent probe 2′,7′-bis-(2-carboxyethyl)-5-(and 6-)-carboxyfluorescein succinimidyl ester (BCECF-AM) (Beyotime Institute of Biotechnology, China) [11]. Firstly, cells during different periods were harvested by centrifugation at 14,972*g* for 10 min. Then the cell pellets were resuspended in PBS buffer (50 mM K_2_HPO_4_, 50 mM KH_2_PO_4_, pH 7.0), washed twice and diluted to an OD_600_ of 3.0. Secondly, 400 µL of the above bacterial suspension and 4 µL valinomycin were added to brown tubes and incubated at 30 °C for 30 min. Thirdly, 1 µL of BCECF-AM was added into the brown tubes and incubated at 30 °C for 20 min; then 200 µL of the reaction solution was taken out and centrifuged at 14,972*g* for 5 min. Lastly, 150 µL of the reaction solution and the supernatant were taken out to measure the fluorescence intensity. Measurements of the fluorescence intensity were performed using a Cytation 3 imaging reader system (BioTek, Winooski, VT, USA). The excitation wavelengths were 490 and 440 nm. The emission wavelength was 525 nm. The relative fluorescence intensity (RFI) was calculated as follows: RFI = [(*I*_490_)_total_ − (*I*_490_)_supernate_]/[(*I*_440_)_total_ − (*I*_440_)_supernate_]. Based on the values of lg (RFI), the intracellular pH was calculated from the standard curve. Measurements were performed with three biological replicates.

### Random mutagenesis of *Ce*GNA1 with Ep-PCR

Ep-PCR was performed using a GeneMorph II Random Mutagenesis Kit (Agilent Technologies, Santa Clara, CA, USA). Mutagenic amplifications were conducted by two separate processes to optimize the quantities of template (0.1, 1, 10, or 100 ng) and the number of amplification cycles (15, 20, 25, and 30). After process optimization, we found that 1 ng of template and 20 cycles of amplification were suitable for the production of one or two amino acids containing mutants. After the amplification under the suitable conditions using the primer pair er-ceN-F1/er-ceN-R1, the PCR products were purified and ligated with the linearized plasmid pP43-cMyc (M-Rm)-*Ce*GNA1 [6], which had been PCR amplified using the primer pair er-ceN-F2/er-ceN-R2 to remove the wild type *cegna1* gene. The ClonExpress™ II kit (Vazyme Biotech Co., Ltd) was used for the ligation, and then the ligation products were used to transform *Escherichia coli* JM109 cells. The resulting colonies growing on the plates were washed down with sterile water, inoculated into LB liquid medium and then cultured for 8 h before plasmid DNA were extracted. Then, the plasmid DNA were transformed into the engineered host strain BSGN12. Preliminary screening of high-yield mutants was conducted in a 96-well plate, using the Reissig method [12]. Finally, the high-yield mutants were confirmed for shake flask fermentation. The mutagenesis selection process is shown in Additional file 1: Fig. S1.

### Purification and activities determination of *Ce*GNA1 and its mutant

For the purification of *Ce*GNA1, the wild type *cegna1* gene was amplified from the plasmid pP_*43*_-6His-*Ce*GNA1 using the primer pair HisCeN-F/HisCeN-R, and then ligated with the expression plasmid pCold III (linearized by PCR amplification with the primer pair pCold-F/pCold-R) using the ClonExpress™ II kit (Vazyme Biotech Co., Ltd), yielding pCold-*Ce*GNA1. Then pCold-*Ce*GNA1 was used as the template to generate pCold-*Ce*GNA1-Q155V/C158G using the one-step site-directed plasmid insertion protocol [13]. The primers used were Q155V/C158G-F and Q155V/C158G-R.

Strains expressing of pCold-*Ce*GNA1 or pCold-*Ce*GNA1-Q155V/C158G were cultured in LB medium, and protein expression was induced by the addition of 1 mM isopropyl-*β*-d-thiogalactopyranoside (IPTG) following a temperature downshift from 37 to 15 °C. After cultivation for 24 h following induction, the strains were harvested by centrifugation at 6000×*g* for 10 min, lysed by sonication on ice, resuspended in 50 mM Tris–HCl buffer (pH 7.5), and then purified via nickel affinity using a Ni^2+^ column [14]. The eluted His_6_-tagged protein was dialyzed against 50 mM Tris/HCl (pH 7.5) and 5.0 mM MgCl_2_, and its purity was confirmed by sodium dodecyl sulfate polyacrylamide gel electrophoresis (SDS-PAGE). The SDS-PAGE was performed as described in Additional file 1: Fig. S3. No denaturants were added before the SDS-PAGE. The reductant dithiothreitol (DTT) added was 30 μM. The protein concentration was determined using the Bradford assay with BSA as standard.

*Ce*GNA1 activity was determined using the 5,5′-dithiobis (2-nitrobenzoic acid) (DTNB) method by measuring the amount of free thiol groups generated during acetyl transfer in Tris–HCl buffer (pH 7.5, 50 mM) [4]. A control without the addition of enzyme was used. The amount of CoASH produced was calculated based on *E *=* εlc* with *ε*^DTNB^ = 137,000 M^−1^ cm^−1^ and *l *= 1 cm. One unit of GNA1 activity was defined as the amount of enzyme that produced 1 nmol of CoASH per minute under the analysis condition. To determine pH stability of *Ce*GNA1, the wild-type and mutant enzymes were incubated in Tris–HCl buffer (pH 5.5 to 7.5, 50 mM) at 30 °C for 12 h. Then the *Ce*GNA1 activity was measured at pH 7.5. For kinetics, 50 ng of enzyme was used, and the GlcN-6P concentrations were 50, 100, 150, 200, 500, 1000, and 2000 μM.

### Expression of urease from *Bacillus paralicheniformis*

The urease gene cluster was integrated at *yoqM* loci, which is a nonessential gene in *B. subtilis* according to *Subti*Wiki (http://subtiwiki.uni-goettingen.de/), and inactivation of it could improve the yield and purities of poly-histidine tagged protein produced in *B. subtilis* according to the US patent WO2016050680A1 [15]. The urease gene cluster *ureABCEFGDH* was amplified from the genomic DNA of *B. paralicheniformis* using the primer pairs ure-F(Pveg)/ure-R, ure-F(PxylA)/ure-R or ure-F(PabrB)/ure-R, respectively [16]. The promoters P_*veg*_, P_*xylA*_, P_*abrB*_, P_*hag*_, P_*ffh*_, and P_*licH*_ were amplified from the plasmid pP_*veg*_*EcGlmS*-1, pStop1622, and the genomic DNA of *B. subtilis* 168, respectively [17]. Then, the front and back homology arms, amplified from the genomic DNA of *B. subtilis* 168, and the zeocin resistance gene, amplified from the plasmid p7Z6, were fused with the corresponding promoters and urease coding genes in the order of *yoqM* (L)-zeo-promoter-*ureABCEFGDH*-*yoqM* (R) using overlap extension PCR [18]. Finally, integration of the fusion products into the chromosome of BSGN12 was conducted as described before [19]. The primer pairs ure 1-F/ure 1-R and ure 2-F/ure 2-R were used in colony PCR for selecting the correct mutants.

### Fed-batch fermentation in a 3-L bioreactor

Fed-batch fermentation of BSGN13, transformed with the plasmid pP43-cMyc (M-Rm)-*Ce*GNA1-Q155V/C158G, was performed in a 3-L fermenter (BioFlo115, New Brunswick Scientific Co., Edison, NJ, USA). Ninety milliliters of seed culture that were cultured in 500-mL flasks for 8–10 h was added to the 3-L fermenter with an initial 1.7 L of fermentation medium. The pH was maintained at 7.3 automatically via the addition of ammonium hydroxide (50% [vol/vol]), and the temperature was maintained at 37 °C. The aeration and agitation rates were 1.5 vvm and 800 rpm, respectively. The initial glucose concentration was 40 g/L, and during fermentation it was maintained at 2–10 g/L using an automatic glucose analyzer.

### Analytical methods

The concentration of urea in the fermentation medium was quantified by high-pressure liquid chromatography with fluorescence detection after automated derivatization with xanthydrol [20]. The concentration of glucose, GlcNAc, and pyruvate in the fermentation broth was analyzed by HPLC as described before [6]. Cell growth was monitored by measuring the absorbance at 600 nm (OD_600_). The correlation between OD_600_ and dry cell weight (DCW) was OD_600_ of 1 = 0.35 DCW (g/L) [21]. All the experiments were performed independently at least three times.

## Results

### Pyruvate stress decreased pH_ex_ and pH_in_

**Fig. 2:**
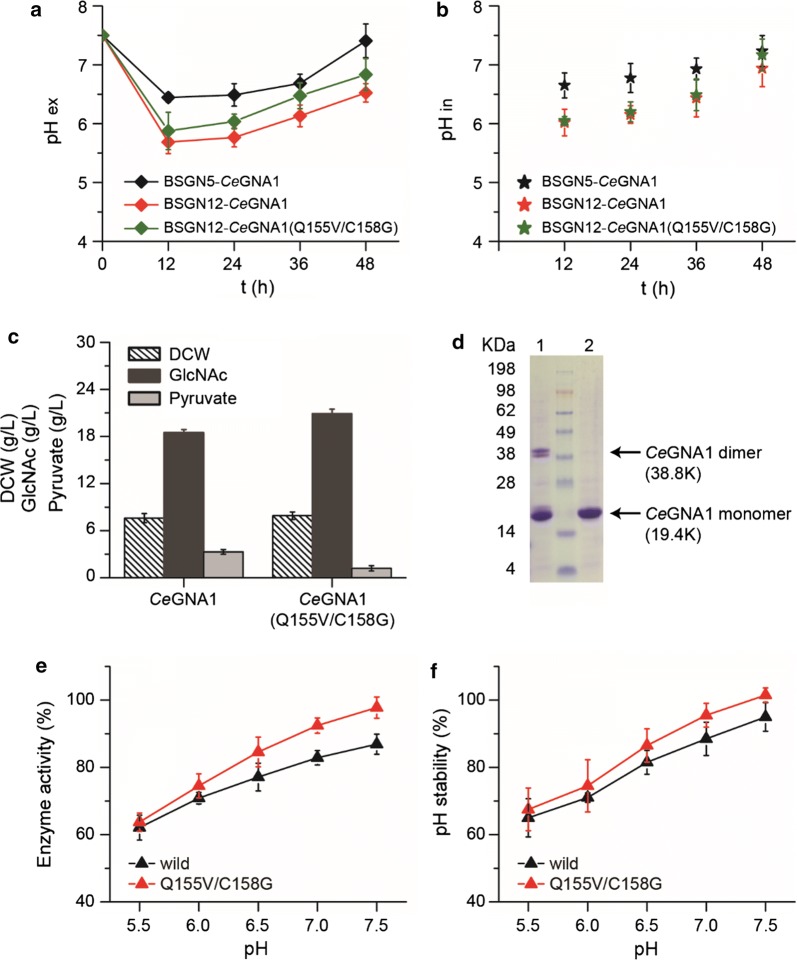
Effects of pyruvate stress and *Ce*GNA1 mutation on GlcNAc fermentation. Comparison of extracellular pH (pH_ex_) (**a**) and intracellular pH (pH_in_) (**b**) during fermentation of the control strain BSGN5 and the engineered BSGN12 transformed with the plasmid pP_*43*_-cMyc (M-Rm)-*CeGNA1* or pP_*43*_-cMyc (M-Rm)-*CeGNA1*-Q155V/C158G, respectively. **c** Effects of *Ce*GNA1 mutation on cell growth (dry cell weight, DCW), GlcNAc production, and pyruvate accumulation. **d** SDS-PAGE analysis of the purified wild type (1, *CeGNA1*) and mutant *CeGNA1* (2, *CeGNA1*-Q155V/C158G). Effects of *Ce*GNA1 mutation on the activity (**e**) and pH stability (**f**) of *Ce*GNA1

During fermentation of BSGN12, transformed with the plasmid pP_*43*_-cMyc (M-Rm)-*CeGNA1*, pyruvate was produced as an overflow metabolite and lowered the pH_ex_ from the initial 7.5 to around 5.7, which was 0.8 units lower than 6.5 as was observed in strain BSGN5 (Fig. 2a). To confirm the lowering of pH_in_ caused by the pyruvate stress, the pH_in_ of BSGN12 cells was measured using a pH-sensitive fluorescent probe BCECF-AM and found that the pH_in_ varied from 6.0 to 6.9 depending on the cellular state, which was decreased compared to that of strain BSGN5 (ranging from 6.6 to 7.2) (Fig. 2b). Because *Ce*GNA1 has optimum activity in alkaline conditions (pH 7.4–9.7), it was assumed that the decreased pH_in_ caused a decrease of *Ce*GNA1 catalytic efficiency, which limited GlcNAc production.

### Mutations of *Ce*GNA1 enhanced its activity and promoted GlcNAc production

To improve the catalytic efficiency of enzyme *Ce*GAN1 under pyruvate stress, a library of *Ce*GAN1 mutants with an average mutation rate of one or two amino acid changes per protein was generated using Ep-PCR. After screening approximately 10^4^ Ep-PCR clones, 15 mutants with enhanced GlcNAc titer were selected and retested in batch cultures and one evolved mutant, *Ce*GAN1-Q155V/C158G, which increased the GlcNAc titer from 18.5 to 20.9 g/L and decreased the extracellular pyruvate from 3.5 to 1.2 g/L, was identified and further characterized (Fig. 2c). The mutagenesis selection process is shown in Additional file 1: Fig. S1. During fermentation of BSGN12, transformed with the mutant *Ce*GNA1-Q155V/C158G, the lowest pH_ex_ increased to 5.9, which was slightly higher than that before (5.7) (Fig. 2a). Specific activity analysis of *Ce*GNA1 in the lysis supernatant found that it increased by 21.7% to 1060 U/mg [6]. However, the decreased pyruvate concentration had little impact on pH_in_ (Fig. 2b). This increase of pH_ex_ and GlcNAc titer might be due to the enhanced catalytic efficiency of *Ce*GNA1-Q155V/C158G during acidic stress.

To gain further insight into the effects of Q155V/C158G mutations on catalytic efficiency, the mutant enzyme *Ce*GNA1-Q155V/C158G was expressed using an *E. coli* expression system, purified, and verified by SDS-PAGE, and its activity in acidic pH was measured. As shown in Fig. 2d, this size was consistent with the calculated protein mass of 19.4 kDa. Compared with the wild-type *CeGNA1*, the mutant *Ce*GNA1-Q155V/C158G lacked the 38.8 kDa band representing a homodimer, which was probably due to the C158G mutation resulting in the lack of a disulfide bond formed between the two monomers, thereby reducing the thermostability of the homodimer such that *Ce*GNA1-Q155V/C158G can completely denature in the same conditions when preparing SDS-PAGE [4]. Analysis of the activity and pH stability of mutant *Ce*GNA1-Q155V/C158G showed that it increased with the increasing pH and was higher than that of the wild type (Fig. 2e, f). Especially in pH 6.5–7.5, the activity of the mutant protein was 11.5% higher than that of the wild type. Kinetic data analysis showed that the Michaelis constant (K_*m*_ value) of *Ce*GNA1-Q155V/C158G for GlcN-6-P (122 μM) was 12.2% lower than that of the wild type, and the *k*_*cat*_/K_*m*_ of *Ce*GNA1-Q155V/C158G (1.25 s^−1^ μM^−1^) was 27.5% higher than that of *Ce*GNA1 (0.98 s^−1^ μM^−1^) (Table 2). These results indicated that the mutations Q155V/C158G increased the substrate-binding ability and improved its catalytic efficiency.

**Table 2:** Kinetic data of wild-type and Q155V/C158G *Ce*GNA1 for GlcN-6-P

	K_*m*_ (μM)	*k*_cat_ (s^−1^)	*k*_cat_/K_*m*_ (s^−1^ μM^−1^)
*Ce*GNA1	139 ± 9	136 ± 1.1	0.98
*Ce*GNA1-Q155V/C158G	122 ± 6	151 ± 1.5	1.23

Previous studies revealed that formation of two disulfide bonds, one formed between Cys158 from the A and B chains and another between the conserved Cys141 and CoA, inhibited the enzyme activity [4]. The increased activity of mutant *Ce*GNA1-Q155V/C158G might be due to the replacement of Cys158 by Gly158 preventing the formation of the disulfide bonds and relieving the inhibition of enzyme *Ce*GNA1 by CoA. Comparation of the GlcNAc titer in strains expressing *Ce*GNA1 with single Q155V or C158G mutations demonstrated that the single mutation of 155Q to 155V had little effect on GlcNAc production, and the single mutation of 158C to 158G was sufficient for the improved yield of GlcNAc (Additional file 1: Fig. S2). Therefore, it could be speculated that 158Cys was the main factor affecting GlcNAc production. Further saturation mutation of C158 site showed that most of the mutants increased GlcNAc production compared with the wild *Ce*GNA1, and confirmed the conclusion that mutation of Gly158 prevents the formation of the disulfide bonds and relieves the inhibition of enzyme *Ce*GNA1 by CoA (Additional file 1: Fig. S2).

### Expression of urease increased the pH_in_ and promoted GlcNAc production

**Fig. 3:**
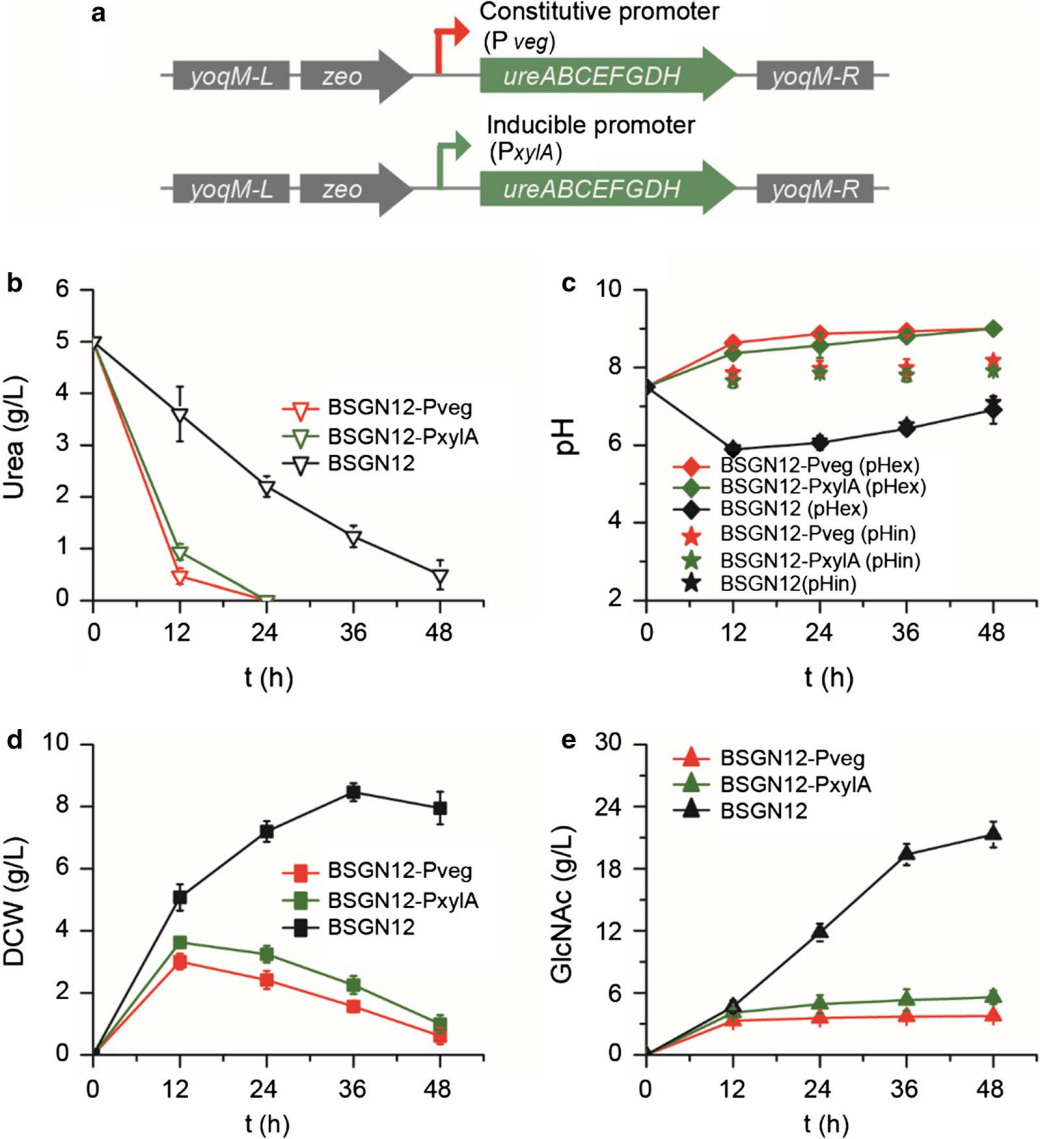
Effects of urease expression on GlcNAc fermentation. **a** Expression of urease were controlled by the constitutive promoter P_*veg*_ and xylose inducible promoter P_*xylA*_, respectively. Effects of urease expression on urea utilization (**b**), pH_ex_ (**c**), cell growth (dry cell weight, DCW) (**d**) and GlcNAc production (**e**)

To generate urease-expressing strains, two urease expression cassettes containing the *ureABCEFGDH* gene cluster from *B. paralicheniformis*, one under the control of constitutive promoter P_*veg*_ and the other under the xylose inducible promoter P_*xylA*_, were integrated into the *yoqM* loci, resulting in BSGN12-P_*veg*_-urease and BSGN12-P_*xylA*_-urease, respectively (Fig. 3a). Shake flask fermentation of BSGN12-P_*veg*_-urease and BSGN12-P_*xylA*_-urease with the plasmid pP43-cMyc (M-Rm)-*Ce*GNA1-Q155V/C158G were conducted in the fermentation medium with 5.0 g/L urea. Expression of urease under the control of promoters P_*veg*_ and P_*xylA*_ strongly promoted urea utilization, with 4.3 g/L urea being consumed during the first 12 h (Fig. 3b). In contrast, urea utilization in the starting strain BSGN12 was slow, with 1.4 g/L urea being consumed during the first 12 h, and a total of 3.6 g/L urea consumed within 48 h. This rapid utilization of urea in BSGN12-P_*veg*_-urease and BSGN12-P_*xylA*_-urease alkalized the culture to a pH of roughly 8.5 (Fig. 3c). Because urease was expressed intracellularly, alkalization of pH_ex_ indicated alkalization of pH_in_ (rising to 7.9), and this limited cell growth as well as GlcNAc production, with the maximum DCW reaching 3.3 g/L and GlcNAc titer being less than 5 g/L (Fig. 3c–e). However, the slow utilization of urea in the starting strain was not enough to counteract the acidification during fermentation, with the decrease of pH_ex_ from 7.5 to 5.9 and the pH_in_ to 6.0, and so could not alleviate the inhibition of pyruvate on the key enzyme *Ce*GNA1 (Fig. 3c).

**Fig. 4:**
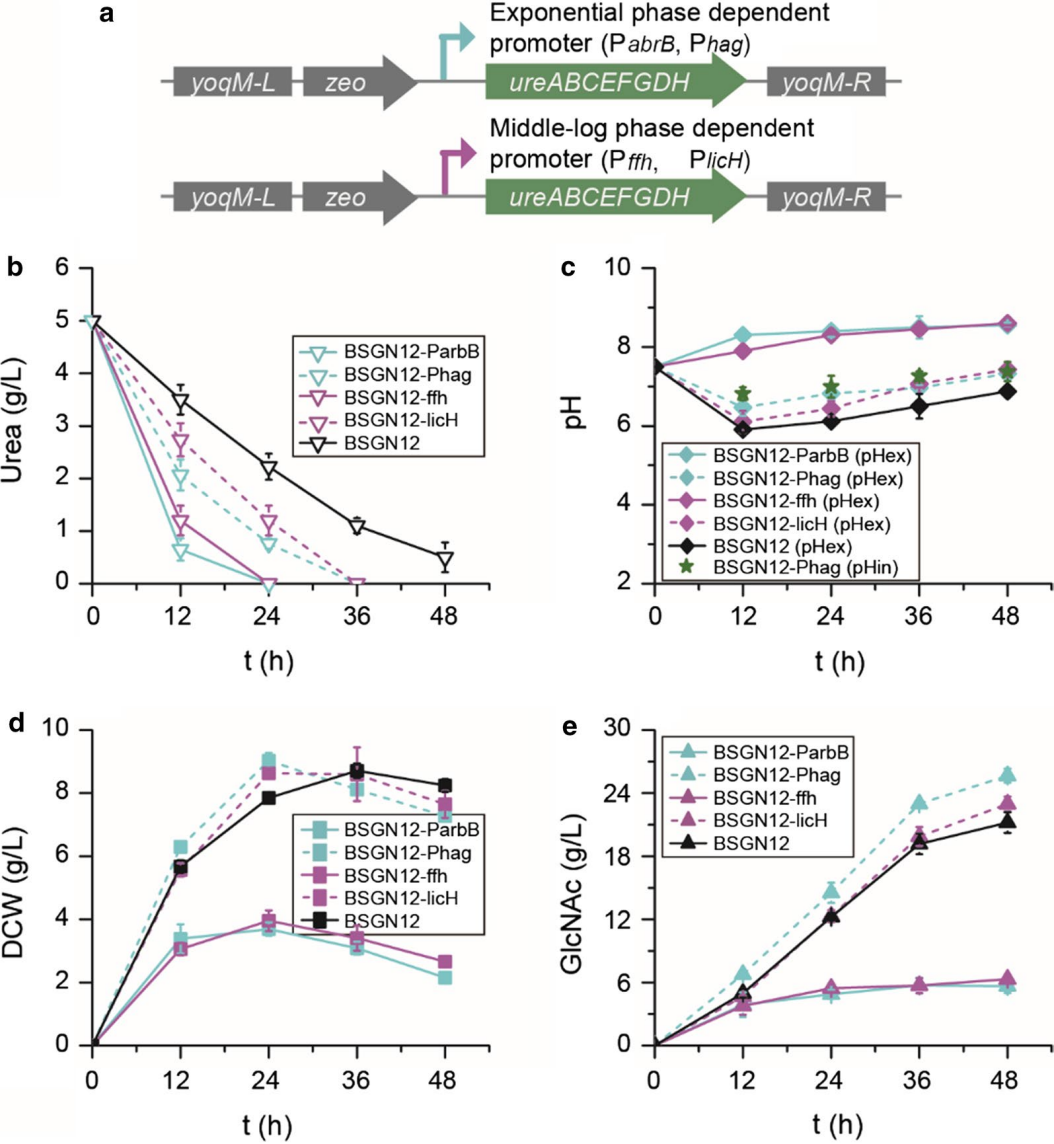
Effects of urease expression on GlcNAc fermentation. **a** Expression of urease were controlled by the exponential phage dependent promoters (P_*abrB*_ and P_*hag*_) and middle-log phage dependent promoters (P_*abrB*_ and P_*hag*_), respectively. Effects of urease expression on urea utilization (**b**), pH (**c**), cell growth (dry cell weight, DCW) (**d**) and GlcNAc production (**e**)

The above results demonstrated that the expression level of urease should be neither too high nor too low. Because a decrease in pH_in_ and pH_ex_ mainly occurred at the early phase of fermentation, the expression of urease should correspond to this phase to alleviate the decrease of pH_in_ and pH_ex_, and then it should be low enough to avoid excessive alkalization during fermentation. To achieve this, two classes of phase-dependent auto-inducible promoters, exponential phase-dependent promoters (P_*abrB*_ and P_*hag*_, with P_*abrB*_ being stronger than P_*hag*_) and middle-log phase-dependent promoters (P_*ffh*_ and P_*licH*_, with P_*ffh*_ being stronger than P_*licH*_), were chosen to control the expression of urease (Fig. 4a) [17].

As shown in Fig. 4, urea utilization depended on the strength and class of the chosen promoters. Consistent with what was described above, strong promoters (P_*abrB*_ and P_*ffh*_), which resulted in rapid utilization of urea and excessive alkalization during fermentation, were not suitable for urease expression and GlcNAc production here. In comparison, the weak promoters (P_*hag*_ and P_*licH*_) were more suitable for urease expression and GlcNAc production, especially the exponential phase-dependent promoter P_*hag*_. Promoter P_*hag*_ promoted the slow utilization of urea, which was consumed within 36 h, and alleviated the decrease of pH_in_, with the lowest pH_in_ increasing from 6.0 to 6.8 (Fig. 4b, c). Meanwhile, the lowest pH_ex_ also increased from 5.9 to 6.4. As a result, the cells grew better, with the maximum DCW 9.0 g/L being 15.2% higher than that of the starting strain BSGN12 at 24 h, and the GlcNAc titer and yield reached 25.6 g/L and 0.43 g GlcNAc/g glucose at the end of the fermentation, which were 1.39- and 1.36-fold of that of the starting strain BSGN12, respectively (Fig. 4d, e).

### Production of GlcNAc by BSGN13 in a 3-L fermenter

**Fig. 5:**
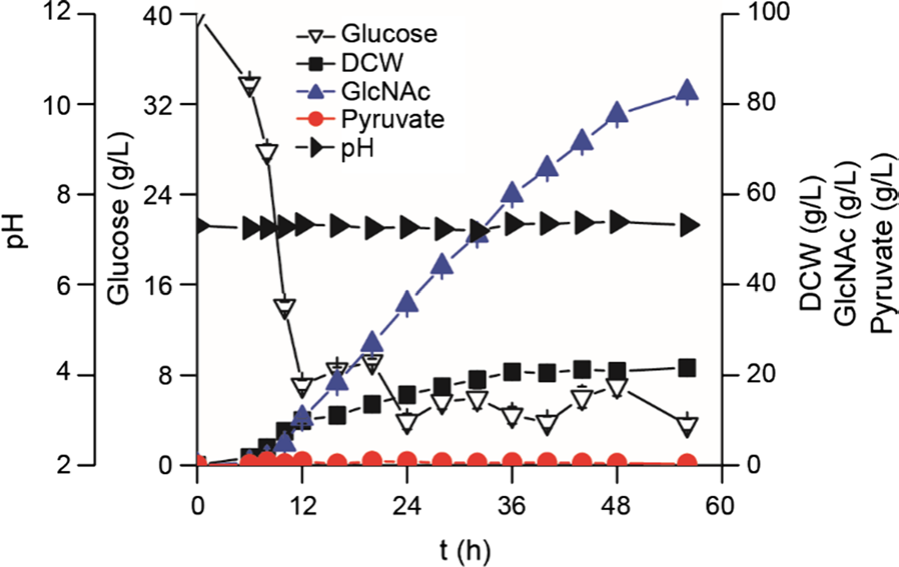
Time profile of fed-batch fermentation of BSGN13 in a 3-L fermenter. In the fed-batch fermentation, inoculation size, temperature, pH, agitation speed, and aeration rate were 5%, 37 °C, 7.3, 800 rpm, and 1.5 vvm, respectively. With the initial concentration being 40 g/L, glucose concentration was maintained at 3–10 g/L using the automatic glucose analyzer during the fermentation. DCW: dry cell weight

As shown in Fig. 5, there was no overflow of pyruvate in the broth during the fed-batch. The engineered *B. subtilis* BSGN13 grew continuously from 0 to 36 h and reached a maximum DCW of 20.7 g/L at 36 h. The GlcNAc in the broth accumulated rapidly along with cell growth and reached 59.8 g/L at 36 h, with an average GlcNAc productivity of 1.66 g/L per hour. Though GlcNAc accumulated gradually from 36 to 56 h and reached 82.5 g/L with a yield of 0.39 g GlcNAc/g glucose, which was 1.7- and 1.2-fold of that produced before (48.9 g/L GlcNAc and 0.32 g GlcNAc/g glucose), the average GlcNAc productivity of 1.13 g/L per hour was obviously decreased [5].

The decreased GlcNAc productivity maybe due to a shortage of nitrogen supply, because even if all the urea and (NH_4_)_2_SO_4_ in the medium were converted to GlcNAc by 100%, only 56.8 g/L GlcNAc could be synthesized, which was 65% of 82.5 g/L. This indicated that most of the nitrogen in GlcNAc originate from yeast extract (12 g/L) and tryptone (6 g/L). Actually, lots of ammonia from urea was used to neutralize the pH_in_, and much of the yeast extract and tryptone were used for cell growth. In the future, we will further study the supply and balance of nitrogen sources to promote GlcNAc production.

## Discussion

It is well known that pyruvate is synthesized from Fru-6P through Embden–Meyerhof–Parnas pathway, and further converted to Ac-CoA. Since synthesis of GlcNAc consumes Fru-6P and Ac-CoA, which competes with pyruvate synthesis and promoting pyruvate utilization, it was speculated that the overflow of pyruvate indicated the synthesis pathway of GlcNAc was not strong enough to divert carbon flux from pyruvate. To enhance the synthesis pathway of GlcNAc and promote GlcNAc production, the two key enzymes *Ce*GNA1 and glucosamine-6-phosphate synthase (GlmS) were previously overexpressed, which decreased pyruvate concentration significantly [6]. However, the residual pyruvate dropped the pH_in_, inhibiting the *Ce*GNA1 activity. Hence, for alleviating pyruvate burden and enhancing GlcNAc production in this study, the key enzyme *Ce*GNA1 was further modified to increase its catalytic efficiency.

A lot of effort has been made to engineer pathway enzymes in metabolic engineering for improved production of value-added biological chemicals [22–24]. Among them, Ep-PCR based directed evolution is a powerful strategy for adapting enzyme properties to specific needs. In the study, Ep-PCR mutation of the *cegna1* gene conferred it enhanced acid-resistance. Because the wild-type *Ce*GNA1 has a pH optimum in the alkaline range (8.2), its activity under acid conditions was low, so we looked for acid resistant forms of the enzyme to improve the yield of GlcNAc. Indeed, owing to limited catalytic performance, the enzymes harvested from nature’s biodiversity often need to be improved for their desired functions. This study also highlighted the importance of pathway protein engineering to efficiently produce value-added biological chemicals in microbial factories.

Although mutations of the key enzyme *Ce*GNA1 improved its catalytic efficiency during pyruvate stress and promoted GlcNAc production in this study, during this process, we found that the commonly used plasmid-based expression systems were prone to genetic instability. For high-level, genetically stable expression of the key enzyme *Ce*GNA1, it is interesting to construct a plasmid-free, high gene copy expression system for GlcNAc production in the future. Maybe integration of *Ce*GNA1 into the genome and regulation of the ratio of *Ce*GNA1 to GlmS, two key enzymes catalyzing two consecutive reactions within GlcNAc synthesis pathway, using artificial protein scaffolds are beneficial for *Ce*GNA1 expression and enhanced metabolic flux channeling to promote GlcNAc production [1, 25].

It has been reported that urease, which catalyzes the hydrolysis of urea to two molecules of ammonia and one molecule of carbon dioxide, plays important roles in maintaining pH_in_ homeostasis and providing ammonium for nitrogen metabolism, which are widely used in metabolic engineering and synthetic biotechnology [26, 27]. Though *B. subtilis* contains urease structural genes, it lacks the accessory genes typically required for GTP-dependent incorporation of nickel, which is essential for urease maturation [28, 29]. For those reasons, urease from *B. paralicheniformis* was heterologously expressed here [16]. Consistent with previous reports where the urease overexpressing *Saccharomyces cerevisiae* strain grew 30–50% slower than the control strain, the strong promoter-controlled expression of urease herein remarkably inhibited BSGN12 growth [27]. However, Milne et al. [27] reported that the cell growth decrease was probably due to high expression of urease accessory enzymes, which led to an increased protein burden, and/or interference with metal metabolism and homeostasis or protein folding. Herein, we demonstrated that it was likely due to ammonia release due to the overexpression of urease, which alkalized the pH_in_ and pH_ex_. Of course, there might have been other factors that also contributed to the cell growth decrease.

## Conclusions

Here, we described mutations of the key enzyme *Ce*GNA1 and heterologous expression of urease from *B. paralicheniformis* to counteract the pyruvate stress for GlcNAc production. The Q155V/C158G mutations enhanced the activity of *Ce*GNA1 by 11.5% and increased the catalytic efficiency by 27.5%, making *Ce*GNA1-Q155V/C158G a promising candidate for GlcNAc production, with the GlcNAc titer increasing to 20.9 g/L in shake flask fermentation. Urease expression under the control of the exponential phase-dependent promoter P_*hag*_ increased pH_in_ from 6.0 to 6.8, relieved acid stress on key enzyme *Ce*GNA1, and increased the titer and yield of GlcNAc to 25.6 g/L and 0.43 g GlcNAc/g glucose, respectively. Finally, in a 3-L fermenter, there was no pyruvate overflow, and the GlcNAc titer reached 82.5 g/L, which was 1.7-fold of that produced before. It was recognized that the pathway enzyme engineering and host engineering regarding urea metabolism were of particular importance to overcome pyruvate overflow for achieving high biosynthesis efficiency of GlcNAc.

## Additional file

**Additional file 1: Table S1.** Primers used in this study. **Fig S1.** The mutagenesis selection process. **Fig S2**. Comparison of the activities of strains with single Q155V and C158G mutants and saturation mutagenesis of C158. **Fig S3.** Identify the double band around 38-39 KDa on the SDS-PAGE.

## Abbreviations

GNA1: glucosamine-6-phosphate *N*-acetyltransferase
GlcNAc: *N*-acetylglucosamine
*B. subtilis*: *Bacillus subtilis*
*Ce*GNA1: GNA1 from *Caenorhabditis elegans*
pH_in_: intracellular pH
Ep-PCR: error prone PCR
GlcNAc-6P: GlcNAc-6-phosphate
GlcN-6P: glucosamine-6-phosphate
