# Supplementary material for: Combinatorial pathway enzyme engineering and host engineering overcomes pyruvate overflow and enhances overproduction of N-acetylglucosamine in Bacillus subtilis
Source: Microb Cell Fact. 2019 Jan 4;18:1. doi: 10.1186/s12934-018-1049-x (PMC6318901; doi:10.1186/s12934-018-1049-x)
Supplement: Supplementary file 1 — Additional file 1: Table S1. Primers used in this study. Fig S1. The mutagenesis selection process. Fig S2. Comparison of the activities of strains with single Q155V and C158G mutants and saturation mutagenesis of C158. Fig S3. Identify the double band around 38-39 KDa on the SDS-PAGE. [file 12934_2018_1049_MOESM1_ESM.docx]

**Table S1**

Primers used in this study.

| Primer | Sequence |
| --- | --- |
| *Random mutagenesis of CeGNA1* | |
| er-ceN-F1 | ggtaccattataggtaagagaggaatgtacacatg |
| er-ceN-R1 | ctatgaccatgattacgccaagctttta |
| er-ceN-F2 | gtaatcatggtcatagctgtttcctgtg |
| er-ceN-R2 | tacctataatggtaccgctatcactttatattttac |
| *Expression and Purification of CeGNA1 in E. coli* | |
| pCold-F | ttatgacccagcgcttttaggtaatctctgcttaaaagcacagaatc |
| pCold-R | ggtggtggtggtggtgcatggtgtattacctcttaataattaagtgtg |
| HisCeN-F | atgcaccaccaccaccaccacagccatatc |
| HisCeN-R | aaagcgctgggtcataaaattacagtcatc |
| Q155V/C158G-F | GGCTTTGTGGATGACTACAATTTTATGACCC |
| Q155V/C158G-R | GGGTCATAAAATTGTAGTCATCCACAAAGCC |
| *Expression of urease* | |
| ure-L-F1 | gtagcagcatcatacttaacgatccattctcc |
| ure-L-F2 | gccttcaaaccaaacccttactcttgttccg |
| ure-L-R | cctgtgtgaaattgttatccgctcccatctccatttccttttaatgtattgcaattac |
| ure-zeo-F | aaggaaatggagatgggagcggataacaatttcacacaggaaacagct |
| ure-zeo-R(Pveg) | GTCAATAAAATTATTTTGACAAAATTagggttttcccagtcacgacgttgtaaaac |
| ure-Pveg-F | cgtcgtgactgggaaaaccctAATTTTGTCAAAATAATTTTATTGACAAC |
| ure-Pveg-R | cacgcggtaatagttgcatGTTTGTCCTCCTTATTAGTTAATCTTTTCTCC |
| ure-F(Pveg) | GATTAACTAATAAGGAGGACAAACatgcaactattaccgcgtgaagtagacaagc |
| ure-R | ggggggaacaccccctcgataaattaaatccaaaggttaaataaaccctcattaatacc |
| ure-R-F1 | tttaacctttggatttaatttatcgagggggtgttcccccctctttcatttttaggag |
| ure-R-F2 | gtttatttaacctttggatttaaggtatgtagggaagtaccaagcaaattattga |
| ure-zeo-R(PxylA) | gtgaacgcaaaggttagcaaaagggttttcccagtcacgacgttgtaaaac |
| ure-PxylA-F | cgtcgtgactgggaaaacccttttgctaacctttgcgttcacttaactaac |
| ure-PxylA-R | cacgcggtaatagttgcatactagtttggaccattgtacatttccc |
| ure-F(PxylA) | gggaaatgtacaatggtccaaactagtatgcaactattaccgcgtgaagtagacaagc |
| ure-zeo-R(PabrB) | CAGGGCGTATGTATATATTagggttttcccagtcacgacgttgtaaaac |
| ure-PabrB-F | cgtcgtgactgggaaaaccctAATATATACATACGCCCTGAAAAAGAATAATT |
| ure-PabrB-R | GTGTACATTTCACCTCCTTTATCCTTACCTTCATAGCATAACAAG |
| ure-F(PabrB)共 | AAAGGAGGTGAAATGTACACatgcaactattaccgcgtgaagtagacaagc |
| ure-zeo-R(Phag) | CCGCAATCACTTTTACTTCAagggttttcccagtcacgacgttgtaaaac |
| ure-Phag-F | cgtcgtgactgggaaaaccctTGAAGTAAAAGTGATTGCGGTTGAAGG |
| ure-Phag-R | GTGTACATTTCACCTCCTTTGAATATGTTGTTAAGGCACGTCCTTGTGC |
| ure-zeo-R(Pffh) | GGAGGGGTATACGGTAAAagggttttcccagtcacgacgttgtaaaac |
| ure-Pffh-F | cgtcgtgactgggaaaaccctTTTACCGTATACCCCTCCATCTGTGC |
| ure-Pffh-R | GTGTACATTTCACCTCCTTTAGTCAAATCCCTTTACGATAACTTAG |
| ure-zeo-R(PlicH) | GAGCGTACAAACagggttttcccagtcacgacgttgtaaaac |
| ure-PlicH-F | cgtcgtgactgggaaaaccctGTTTGTACGCTCAAAAAACCGATCGATTGG |
| ure-PlicH-R | GTGTACATTTCACCTCCTTTGCTGTTGCTATCATCATATTATGAAATCG |
| ure-1F | ctcctgaaataacggtcgatccacaaac |
| ure-1R | ctcgtttgaattgaagcgcttcattcgc |
| ure-2F | ggttttccgtacgggtattagcaaatc |
| ure-2R | cgtttttatggcggcgcttatggtcg |


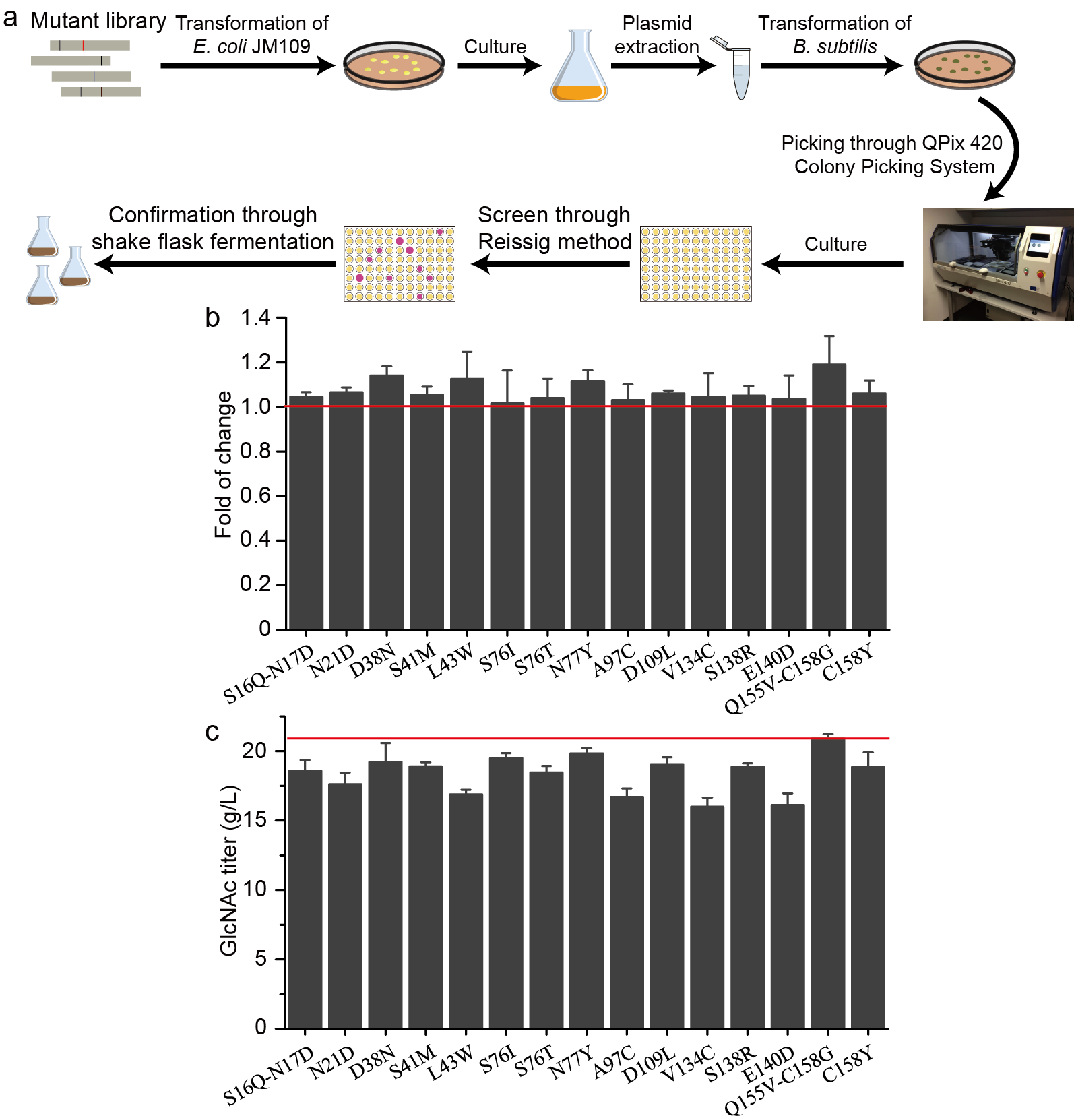


Fig. S1 The mutagenesis selection process.

**(a)** The mutagenesis was selected based on the Reissig method using a BioTek-Cytation 3 multi-format Microplate reader (BioTek Instruments, Inc., Winooski, VT, USA). **Firstly**, the mutants were cultured in 96-well deep plates for 24 h at 37℃, then the strain cultures were centrifuged at 4713 g (Allegra X-15R Centrifuge, Beckman Coulter, United States) for 5 min and 10 μL supernatants were transferred to a new 96-well PCR plate. **Secondly**, 2 μL of 240 mM aqueous solution of potassium tetraborate (pH = 9.2) was mixed with the supernatants, and heated at 98℃ (Applied biosystems, Thermo Fisher Scientific, United States) for 3 min. **Thirdly**, 60 μL 4-Dimethylaminobenzaldehyde (PDABA) solution was mixed with the above 12 μL mixture, and heated at 37℃ for 20 min. **Lastly**, the absorbance was measured at 585 nm using the BioTek-Cytation 3 multi-format Microplate reader. For the configuration of PDABA solution, ten grams of PDABA was diluted in a mixture of 25 mL HCl and 975 mL acetic acid.

**(b)** Based on the absorbance, 15 mutants were selected and confirmed through shake flask fermentation, among which the GlcNAc titer of mutant Q155V/C158G was the highest.

**(c)** Retest of the 15 mutants by shake flask fermentation. The red line corresponds to the maximum value achieved with these mutants.


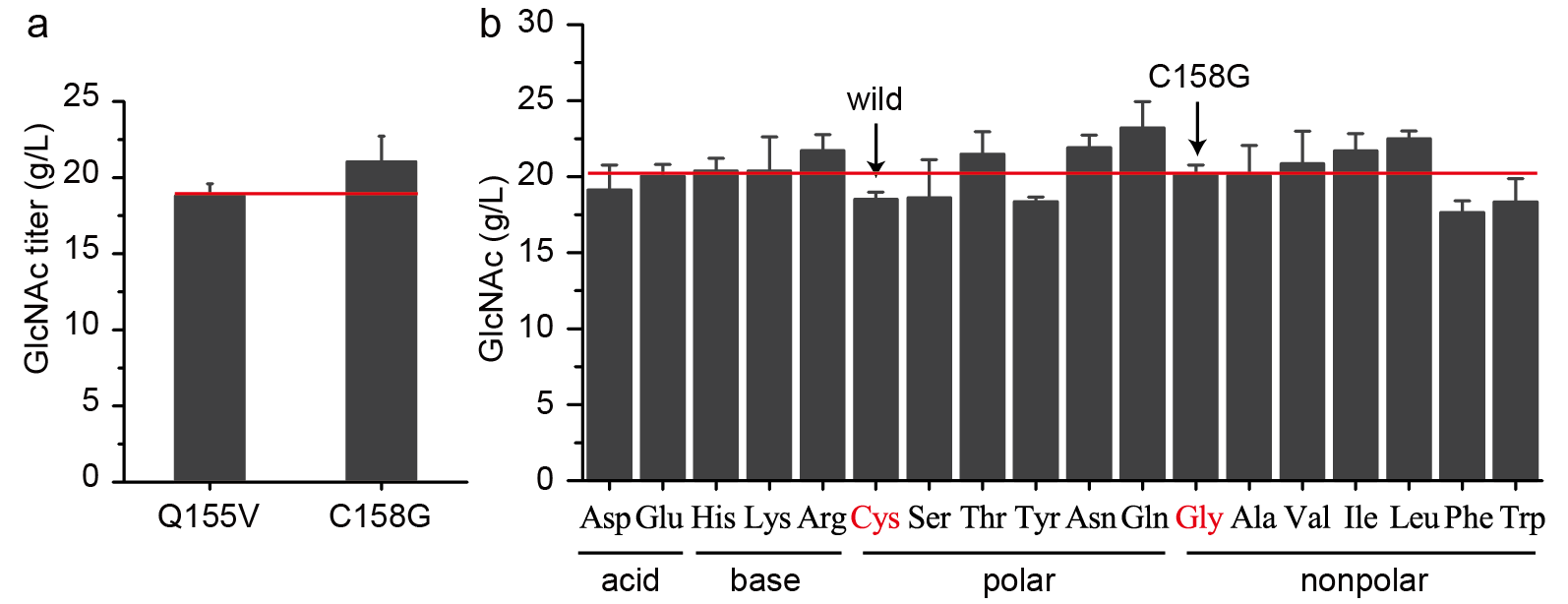


Fig. S2 Comparison of the activities of strains with single Q155V and C158G mutants and saturation mutagenesis of C158.

**(a)** To see whether Q155V or C158G is sufficient for the improved activity/yield of GlcNAc, we firstly compared the GlcNAc titer of Q155V and C158G. As shown in Fig. S2 a, the single mutation of 155Q to 155V had little effect on GlcNAc production, and the single mutation of 158C to 158G was sufficient for the improved yield of GlcNAc. Therefore, we speculated that 158Cys was the main factor affecting GlcNAc production. The red line corresponds to the value achieved with wild-type.

**(b)** Further to see whether C158 change have to be to a small amino acid, the saturation mutation of C158 was conducted. As shown in Fig. S2 b, the C158 change there was no need to be a small amino acid, because C158R, C158N, C158Q and C158L, which had a larger side chain, also increased GlcNAc production, and were better than C158G. The red line corresponds to the value achieved with C158G single mutant. Fortunately, in the process of saturation mutation, we found mutant C158Q performed best, with GlcNAc titer reaching 23.2 g/L. Based on the studies of Ep-PCR, double, triple, and quadruple mutants of *Ce*GNA1 are constructed conducted on the way to further improve its catalytic efficiency, and these results will be presented in the future.


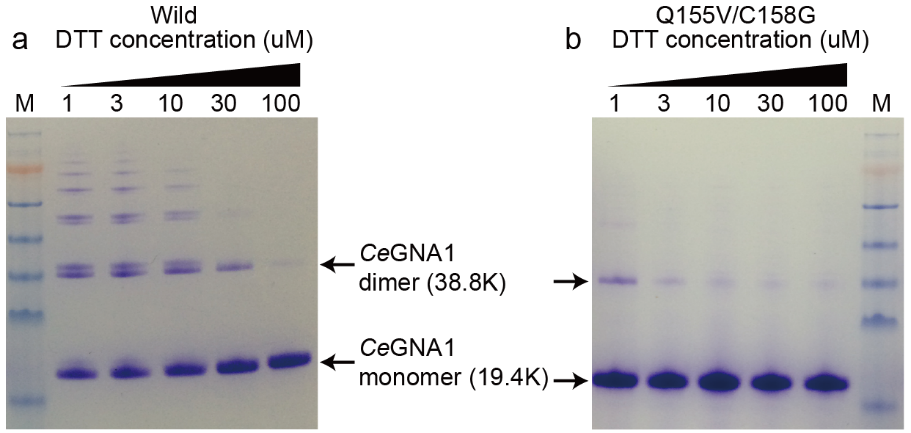


Fig. S3 Identify the double band around 38-39 KDa on the SDS-PAGE.

The SDS-PAGE was performed on a NuPAGE^TM^ 10 % Bis-Tris gel with NuPAGE MES SDS running buffer (Invitrogen, Carlsbad, CA, USA). The proteins were stained by Coomassie Brilliant Blue G250. No denaturants were added before the SDS-PAGE. The reductant dithiothreitol (DTT) added was 1-100 μM, after addition of DTT, the samples were heated at 37℃ for 30 min. Before the SDS-PAGE, the sample was mixed with NuPAGE LDS Sample Buffer (4×) (Invitrogen, Carlsbad, CA, USA), and heated at 70℃ for 30 min.

**(a, b)** As can be seen in Fig. S3a, for the wild type *Ce*GNA1, when the concentration of DTT was low, several bands corresponding to *Ce*GNA1 polymers were found on the SDS-PAGE. With the increasement of DTT concentration, these polymers bands disappeared gradually and there was only one band, corresponding to the *Ce*GNA1 monomer, could be found at 100 μM DTT. For the Q155V/C158G mutant (Fig. S3b), when compared with the wild type *Ce*GNA1 under the same concentration of DTT, the polymers bands disappeared. Thus, we speculated that these bands belong to *Ce*GNA1 polymers. The double band for the polymers might be due to some of the wild type *Ce*GNA1, which should be partially denatured under this condition, forming a partially unfolded intermediate, which was also seen in the following reference [1, 2].

1. Zhang K, Wang L, Liu YX, Chan KY, Pang XY, Schulten K, Dong ZY, Sun F. Flexible interwoven termini determine the thermal stability of thermosomes. Protein & Cell*.* 2013; 4:432-44.

2. Gupta S, Yadav S, Suryanarayanan V, Singh SK, Saxena JK. Investigating the folding pathway and substrate induced conformational changes in *B. malayi* Guanylate kinase. International Journal of Biological Macromolecules*.* 2017; 94:621-33.
